# Chromosomal differences between European and North American Atlantic salmon discovered by linkage mapping and supported by fluorescence *in situ* hybridization analysis

**DOI:** 10.1186/1471-2164-13-432

**Published:** 2012-08-28

**Authors:** Silje Brenna-Hansen, Jieying Li, Matthew P Kent, Elizabeth G Boulding, Sonja Dominik, William S Davidson, Sigbjørn Lien

**Affiliations:** 1 Centre of Integrative Genetics and Department of Animal and Aquacultural Sciences, Norwegian University of Life Sciences, P.O. Box 5003,, 1430, Ås, Norway; 2 Department of Molecular Biology and Biochemistry, Simon Fraser University, Burnaby, British Columbia, V5A 1S6, Canada; 3 Department of Integrative Biology, University of Guelph, Guelph, Ontario, N1G 1W8, Canada; 4 CSIRO Food Futures Flagship and Livestock Industries, Locked Bag 1, Armidale, New South Wales, 2350, Australia

## Abstract

**Background:**

Geographical isolation has generated a distinct difference between Atlantic salmon of European and North American Atlantic origin. The European Atlantic salmon generally has 29 pairs of chromosomes and 74 chromosome arms whereas it has been reported that the North American Atlantic salmon has 27 chromosome pairs and an NF of 72. In order to predict the major chromosomal rearrangements causing these differences, we constructed a dense linkage map for Atlantic salmon of North American origin and compared it with the well-developed map for European Atlantic salmon.

**Results:**

The presented male and female genetic maps for the North American subspecies of Atlantic salmon, contains 3,662 SNPs located on 27 linkage groups. The total lengths of the female and male linkage maps were 2,153 cM and 968 cM respectively, with males characteristically showing recombination only at the telomeres. We compared these maps with recently published SNP maps from European Atlantic salmon, and predicted three chromosomal reorganization events that we then tested using fluorescence *in situ* hybridization (FISH) analysis. The proposed rearrangements, which define the differences in the karyotypes of the North American Atlantic salmon relative to the European Atlantic salmon, include the translocation of the p arm of ssa01 to ssa23 and polymorphic fusions: ssa26 with ssa28, and ssa08 with ssa29.

**Conclusions:**

This study identified major chromosomal differences between European and North American Atlantic salmon. However, while gross structural differences were significant, the order of genetic markers at the fine-resolution scale was remarkably conserved. This is a good indication that information from the International Cooperation to Sequence the Atlantic salmon Genome, which is sequencing a European Atlantic salmon, can be transferred to Atlantic salmon from North America.

## Background

Genome duplication and chromosome segment duplications are recognized as some of the most important mechanisms driving evolutionary change and speciation, with new gene functions arising as a result of gene redundancy occurring after a genome duplication [1]. The common ancestor of extant salmonid fishes is thought to have been an autotetraploid generated during a 4^th^ vertebrate genome duplication event (GDE), which took place 25–100 MYA [2,3]. A consequence of this is that the number of chromosome arms (NF) in salmonids generally varies between 96–104 [4] compared to most teleost species, especially freshwater groups, which have a karyotype of 25 pairs of acrocentric chromosomes and NF of 48–52 [5]. The re-diploidization process which occurs after a genome duplication is not well understood, but an increase in the frequency of repetitive elements such as transposons [6] and a reduction in chromosome number through Robertsonian fusions, translocations, pericentric inversions and deletions are thought to be important mechanisms changing the genome architecture [3]. Chromosomal re-arrangements include fusions that change the karyotype by fusing acrocentric chromosomes, fissions that split a metacentric chromosome at the centromere, giving two acrocentric chromosomes and translocations that move part of one chromosome, often an entire arm, to another chromosome [7].

The Atlantic salmon (*Salmo salar*) represents an important model for studying the mechanisms under-lying vertebrate chromosome re-arrangements and re-diploidization because of the recent genome duplication and varying modal chromosomal numbers in populations. The European Atlantic salmon metapopulation extends from Northern Europe to Spain, while the North American Atlantic salmon metapopulation is found in the Northeastern USA and Canada [8]. There is a large body of evidence indicating molecular genetic divergence between European and North American Atlantic salmon [9] including studies of allozymes [10], mini- and micro-satellites [11,12], rDNA polymorphisms [13] and variation in mtDNA [14-18]. Moreover, fish belonging to these two metapopulations show differences in chromosome number and structure, which allow them to be classified as having either a European or North American karyotype [19]. European Atlantic salmon generally have 29 pairs of chromosomes and 74 chromosome arms [20], whereas it has been reported that North American Atlantic salmon have 27 chromosome pairs and an NF of 72 [21]. The European karyotype consists of 8 pairs of metacentric chromosomes together with 7 pairs of small- and 14 pairs of large-acrocentric chromosomes. By contrast, the standard North American karyotype comprises 9 pairs of metacentric chromosomes, 5 pairs of short- and 13 pairs of long- acrocentric chromosomes [22]. The differences in karyotype between the two metapopulations include an additional metacentric chromosome in the North American karyotype, which is most likely derived from a Robertsonian fusion of two acrocentric chromosomes [22]. Also the European chromosome, ssa01, is significantly larger than its North American counterpart and has an extra C-band on the q-arm, indicating a tandem fusion, differentiating the arm into ssa01qa and ssa01qb. A minimum of eight chromosomalrearrangements have been proposed to explain the chromosomal differences between North American and European salmon [22]. In a study based on linkage mapping in a single North American salmon family from the Saint John River, Lubieniecki et al. [23], proposed two Robertsonian fusions; one between European ssa06 and ssa22 and another between European ssa26 and ssa28. However, these suggested chromosomal rearrangements do not fully explain the major karyotype and the difference in size and C-banding pattern of the largest metacentric chromosome.

Recently a customized European Atlantic salmon SNP-array has been developed and used to construct a dense linkage map for European salmon [24]. The genetic map contains 5,650 SNPs positioned on 29 linkage groups, which agrees well with the expected number of chromosome pairs in the European karyotype [25]. In this study we have used an improved version of the same SNP-array to construct a genetic linkage map of Atlantic salmon from North America. The map was built using genotyping data from two distinct populations of North Atlantic origin and positioned markers on 27 linkage groups. As similar SNP-arrays in both studies enabled positioning of markers on both maps, the linkage map data could be used to predict the major chromosomal rearrangements between Atlantic salmon from either side of the Atlantic Ocean. In addition, FISH analysis was performed to test the proposed karyotype differences.

## Results and discussion

### Linkage map construction in North American Atlantic salmon

Female and male genetic linkage maps, consisting of 3,662 SNPs covering 27 chromosomes, were constructed for the North American Atlantic salmon (Additional file 1). Of these, 3,055 SNPs have also been assigned positions within the European Atlantic salmon linkage map [24] with the remaining 607 SNPs being North American specific. The total lengths of the female and male linkage maps were 2,153 cM and 968 cM, respectively. While females showed a relatively even distribution of recombination along the length of each of their chromosomes, recombination was very concentrated towards telomeres in males as previously shown in European Atlantic salmon [24] and other salmonids [26,27]. Synteny and marker order within chromosomal regions were well conserved. Therefore, markers not separated by recombination in the current study were ordered according to the European Atlantic salmon linkage map.

Despite using similar SNP-arrays, far fewer SNPs were integrated into the North American maps as compared to the European map (3,662 compared to 5,650). There are at least three possible reasons for this. First, the majority of the markers included on the SNP-array were developed based on sequence data generated predominantly from European Atlantic salmon, which could introduce ascertainment bias when the SNP-array is used to genotype salmon of North American origin. Second, the majority of the mapping families were from an aquaculture stock that was imported to New South Wales from the River Phillip in Nova Scotia in the 1960s and subsequently introduced to Tasmania in the 1980s [28]. Since this stock was created from a limited base population, and has been kept isolated for more than 30 years, a founder effect may have caused reduced variability in these mapping families [29]. Third, the remaining mapping families came from an aquaculture program that is based on Saint John River Atlantic salmon, and the founders of this farmed population may also have experienced a founder effect.

### Chromosomal rearrangements between European and North American Atlantic salmon

**Figure 1:**
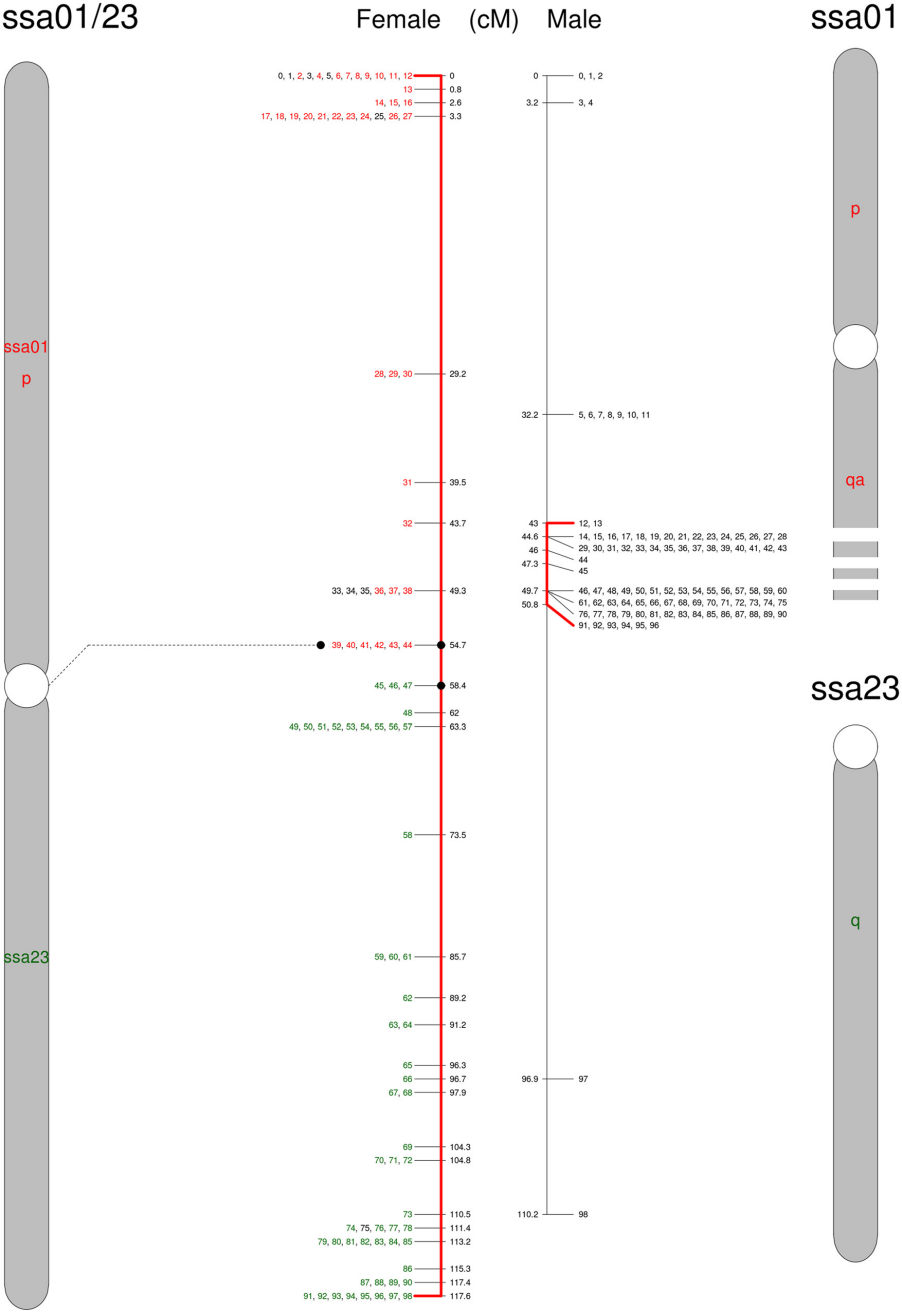
**A translocation involving the European ssa01 p-arm and ssa23 to create a metacentric chromosome in North American salmon (ssa01p/23).** The European ssa01 (red) and ssa23 (green) are shown to the right and the new acrocentric chromosome in North American salmon to the left. Numbers correspond to SNPs listed in Additional file 1. SNPs in colors are shared between Atlantic salmon from both sides of the Atlantic Ocean. Red lines extend to the set of SNPs with limited recombination in the male map.

**Figure 2:**
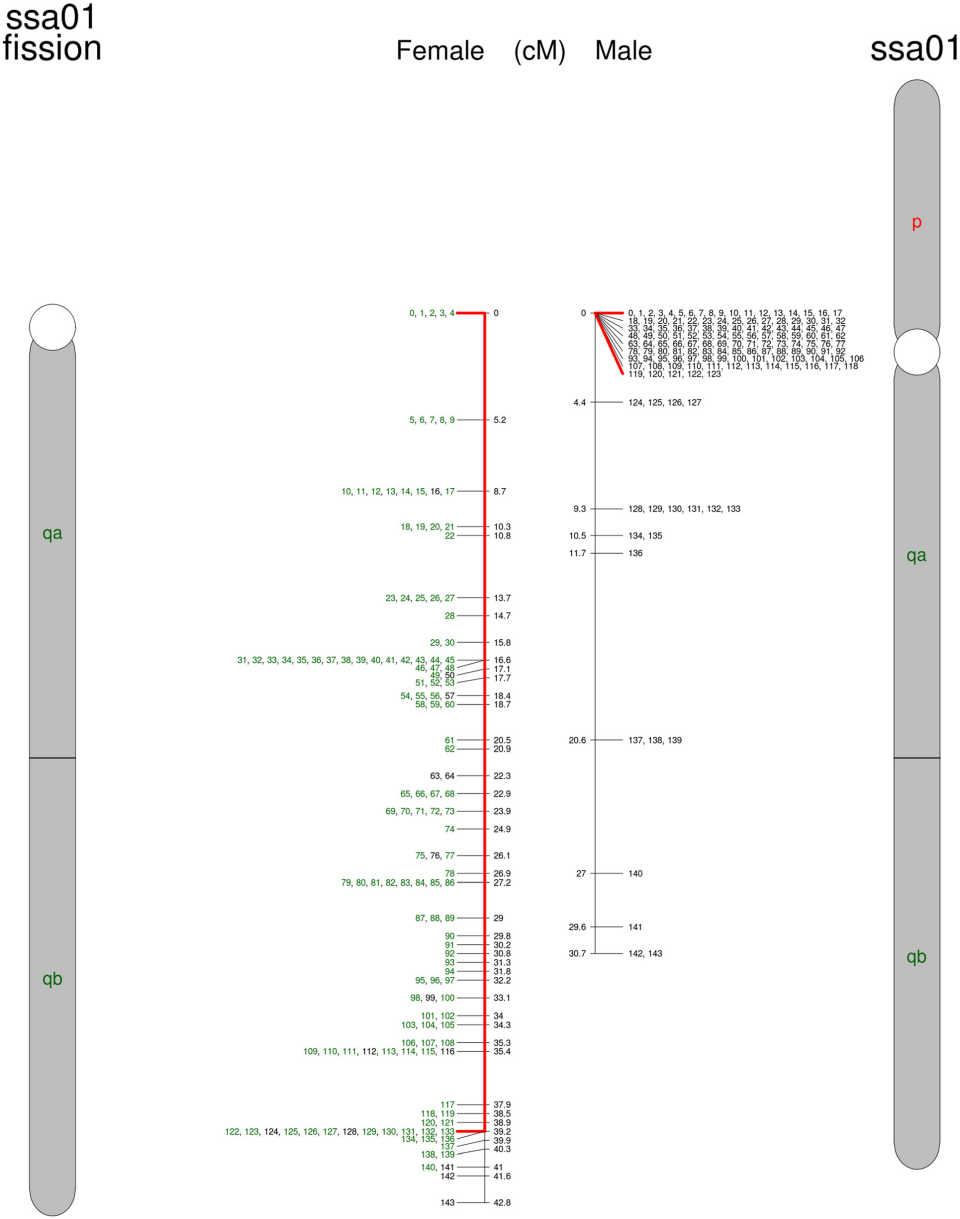
**A chromosomal fission of European ssa01 creating an acrocentric chromosome in North American Atlantic salmon (ssa01q-fission).** The European ssa01 is shown to the right and the new acrocentric chromosome in North American salmon is shown to the left. Numbers correspond to SNPs listed in Additional file 1. SNPs in colors are shared between Atlantic salmon from both sides of the Atlantic Ocean. Red lines extend to the set of SNPs with limited recombination in the male map.

**Figure 3:**
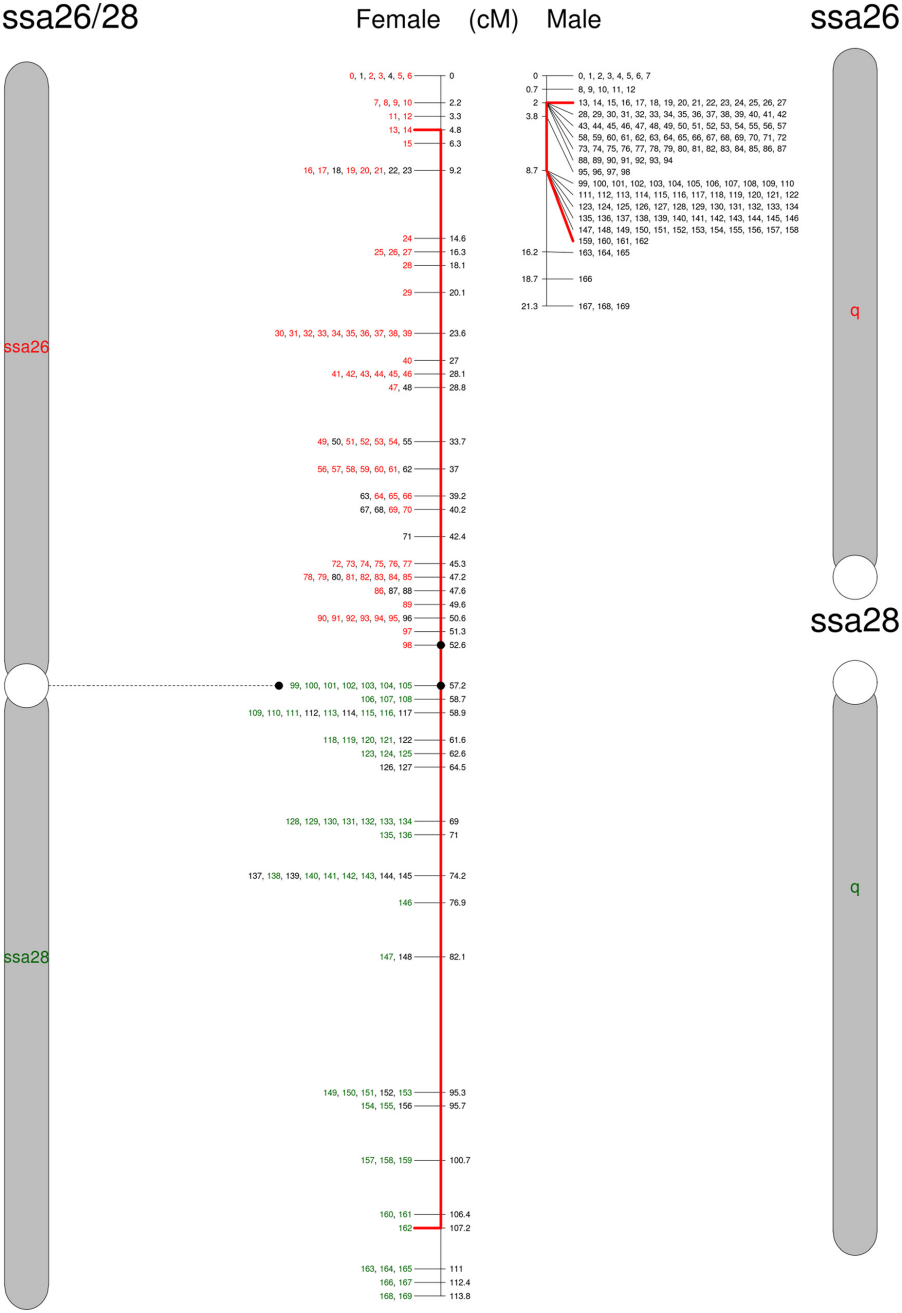
**Genetic map of North American Atlantic salmon chromosome ssa26/28.** The proposed chromosome is to the left and the corresponding European Atlantic salmon chromosome(s) to the right. Numbers correspond to SNPs listed in Additional file 1. SNPs in colors are shared between Atlantic salmon from both sides of the Atlantic Ocean. Red lines extend to the set of SNPs with limited recombination in the male map. The chromosome consists of the two small acrocentric European chromosomes ssa26 (red) and ssa28 (green) fused head to head. The new chromosome ssa26/28 is metacentric.

**Figure 4:**
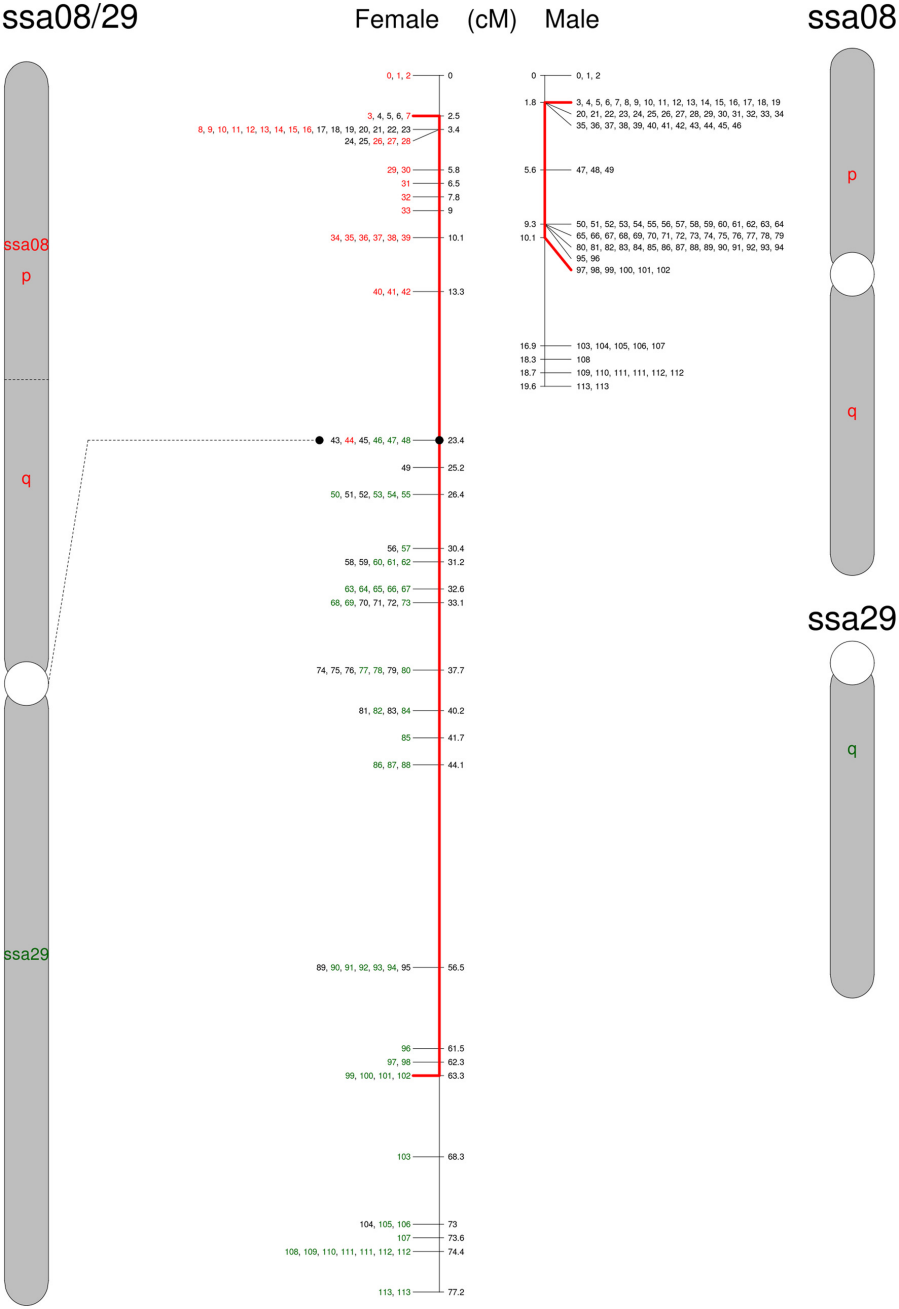
**Genetic map of North American Atlantic salmon chromosome ssa08/29.** The proposed chromosome is to the left and the corresponding European Atlantic salmon chromosome(s) to the right. Numbers correspond to SNPs listed in Additional file 1. SNPs in colors are shared between Atlantic salmon from both sides of the Atlantic Ocean. Red lines extend to the set of SNPs with limited recombination in the male map. The chromosome consists of the metacentric European ssa08 (red) fused with the acrocentric European ssa29 (green) tail to head, causing a predicted centromere shift to the fusion point. The new chromosome ssa8/29 is metacentric.

**Figure 5:**
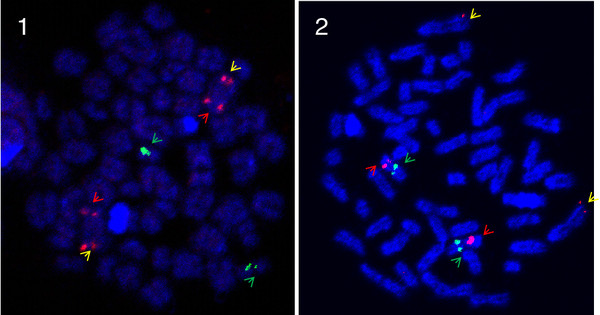
**Ssa01p/23 FISH-images with arrows pointing at BAC probes.** Metaphase spreads of a North American Atlantic salmon from Aqua Bounty Canada and a European Atlantic salmon. Red label with red arrow: BAC S0198E23 on ssa01p. Red label with yellow arrow: BAC S0088O23 on ssa01q. Green label: BAC S0102N22 on ssa23. **1**: European Atlantic salmon: The red signals go to a large metacentric chromosome in the European sample, and the green signal is visible on a small acrocentric. **2**: North American Atlantic salmon: In the North American salmon the red signals are separated. Instead of the large metacentric we see two large acrocentric chromosomes with one red signal, and two smaller metacentric chromosomes with red and green signals.

**Figure 6:**
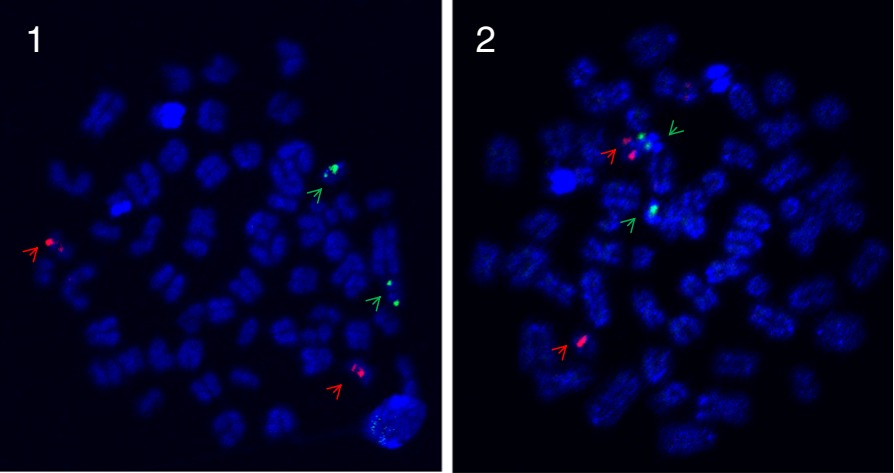
**Ssa26/28 FISH-images with arrows pointing at BAC probes.** Metaphase spreads of a male North American Atlantic salmon from Aqua Bounty Canada and a European Atlantic salmon. Red label: BAC S0059P02 on ssa26, Green label: BAC S0091M08 on ssa28. **1**: European Atlantic salmon: The signals were found on different acrocentric chromosome pairs in the European sample. **2**: North American Atlantic salmon: The signals were joined on a metacentric chromosome in the North American salmon (fusion of ssa26 and ssa 28) and on the two unfused acrocentric chromosomes (ssa26 and ssa28), indicating that this North American Atlantic salmon is heterozygous for the fusion.

**Figure 7:**
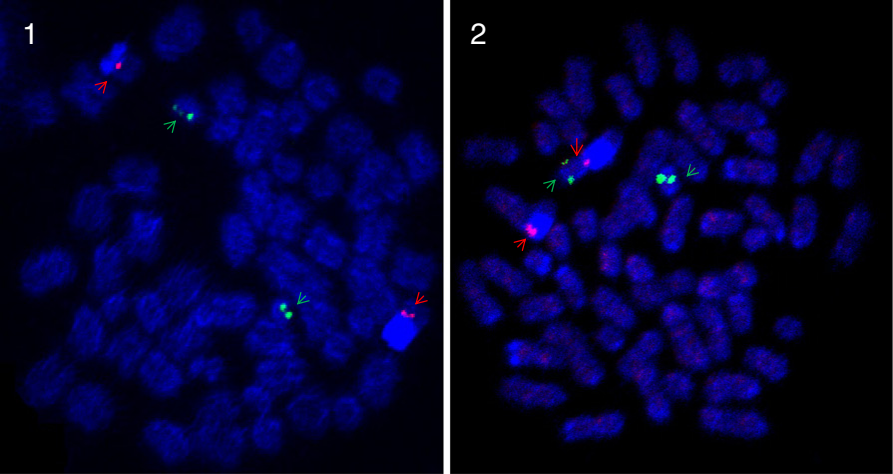
**FISH-images of ssa08/29 with arrows pointing at BAC probes.** Metaphase spreads of a North American Atlantic salmon from Aqua Bounty Canada and a European Atlantic salmon. Red label: BAC S0214J02 on ssa8, Green label: BAC S0016D16 on ssa29 **1**: European Atlantic salmon: As predicted the signals go to individual chromosomes in the European sample. Note the intense light blue stain of heterochromatin on ssa08, which is where the rRNA genes are located. **2**: North American Atlantic salmon: The signals co-locate to separate chromosomes (fusion of ssa08 and ssa29) and also to the two unfused acrocentric chromosomes (ssa08 and ssa29), indicating that this North American Atlantic salmon is heterozygous for the fusion.

The linkage maps constructed for European and North American Atlantic salmon have 3,055 SNPs in common, which allowed us to perform a comprehensive comparison of chromosomal structures between Atlantic salmon from either side of the Atlantic Ocean. This comparison suggests the creation of three composite chromosomes, including a novel metacentric chromosome, in North American Atlantic salmon differing from the European karyotype (Figures 1, 2, 3 and 4). Additional evidence for the proposed chromosomal rearrangements is provided by cytogenetic studies and FISH analyses in Atlantic salmon of European and North American origin (Figures 5, 6 and 7). Although it is likely that the North American and European karyotypes have differentiated in parallel and that one population is not the origin of the other, for the purposes of clarity, the following paragraphs use the European karyotype as the basis with which to explain differences as this is the karyotype with the higher NF.

The first proposed rearrangement involves the European chromosomes ssa01 and ssa23. We suggest that a chromosomal translocation event joined the p-arm of ssa01 to ssa23 (ssa01p/23; Figure 1), leaving behind a new acrocentric chromosome of the ssa01q-arm in the North American Atlantic salmon (ssa01q fission; Figure 2). Taking into account the order of markers and the increased recombination towards the end in male map, we conclude that this is a centromere to centromere fusion. This is also supported by the FISH results. Figure 5 shows a triple FISH experiment where two red probes from opposite ends of ssa01 are detected on the same metacentric chromosome in the European sample, but separated on both acrocentric and metacentric chromosomes in the North American salmon. At the same time, a green probe for ssa23 alone, hybridizes to a single set of acrocentric chromosome pairs in the European sample, but is found together with one of the metacentric red probes in the North American sample. As other cytogenetic studies of North American salmon have mentioned variable numbers of the large metacentric chromosome, this could suggest that spontaneous fission or other mechanisms to stabilize meiosis in hybrids occur. We propose that the two ssa01 derived chromosomes in North Atlantic salmon are named ssa01q and ssa01p/23 according to their origin in European Atlantic salmon. This chromosomal translocation does not introduce alterations in the NF or 2n, but it presents an explanation for the observed difference in size between the first chromosome (ordered by size) in meiotic preparations of the two Atlantic salmon karyotypes shown in [19].

The second proposed major rearrangement involves the small acrocentric European chromosomes ssa26 and ssa28, which in North American salmon appear to have fused in a centromere-to-centromere direction (ssa26/28; Figure 3). This finding is supported by an increased recombination at the ends of the chromosome in the North American Atlantic salmon male linkage map and FISH results. As shown in Figure 6, ssa26 and ssa28 specific probes hybridize to different chromosomes in the European sample; however, North American Atlantic salmon are polymorphic for this fusion, and Figure 6 shows the karyotype of a heterozygote, as the probes are detected together on one chromosome pair (presumably of North American origin) and separately on two others. We named this composite chromosome found in North American salmon ssa26/28 to reflect its relationship to the European Atlantic salmon chromosomes [25]. The formation of the metacentric ssa26/28 reduces the number of acrocentric chromosomes by two relative to the European Atlantic salmon, and introduces a new metacentric chromosome while the number of chromosome arms remains unaltered.

The third proposed rearrangement involves the European chromosomes ssa08 (metacentric) and ssa29 (acrocentric). Although linkage results are conclusive regarding fusion of these two chromosomes in North American Atlantic salmon, it is not easy to deduce the architecture of this chromosome. We hypothesise that ssa29 combined through a tandem fusion with the q-arm of ssa08, preserving the centromere position of ssa8 (Figure 4). This is supported by FISH analysis as seen in Figure 7. It is proposed that the presence of rDNA at or close to the breakage site can stabilize chromosomes that have undergone fission [30]. As can be seen from the FISH imaging, the heterochromatin staining of rDNA genes was present in both the European fish and the North American. It is thus not likely that the rDNA genes on the short p-arm of European Atlantic salmon ssa08 [25] is translocated to another chromosome. We conclude that the North American ssa08/29 is a metacentric chromosome named according to its origin in the European Atlantic salmon linkage map and that the tandem fusion reduces the NF by two from 74 in European Atlantic salmon to the documented 72 in North American Atlantic salmon [21].

The proposed chromosomal rearrangements in the North American Atlantic salmon relative to the European Atlantic salmon described above provide an explanation for the reported differences in karyotype between the two sub-species. However, our results from FISH-analysis also indicated a heterogeneous state of the two fusion chromosomes, suggesting that there are polymorphic variations in the North American karyotype that need to be studied in more detail.

Lubieniecki *et al*. [23] represents the first to attempt to explain the chromosomal rearrangements in European and North American Atlantic salmon by comparative linkage mapping using a map comprising 280 microsatellite markers in a single, relatively small family (NB1) of the Saint John River strain. The authors describe a fusion of ssa26 and ssa28, which is supported by our analysis. However, their suggestion of a fusion between ssa06 and ssa22, is only weakly supported by their data, and was not found in our linkage data. Moreover, FISH analysis provided no indication that their putative ssa06 and ssa22 fusion exists in Atlantic salmon of North American origin (Li and Davidson, unpublished results). The predicted fusions of Lubieniecki *et al.*[23] came from the male map, and it is important to note that these were not seen in the female map. In addition, they did not detect the translocation between ssa01p and ssa23, but as the authors note, their family has relatively limited mapping power and their ssa01 comprise three linkage groups (NB1-17fa, NB1-17fb and NB1-17 fc) in the female map. One of these corresponds to ssa01p, whereas the other two correspond to segments of ssa01q, which has previously been shown to be equivalent to two chromosome arms of rainbow trout [25]. Therefore, their results are not in disagreement with what is reported here; rather, their study reveals the limitations of basing conclusions on linkage maps derived from a single family and containing a relatively small number of genetic markers. Evidently, the much larger dataset in the present study, which involved thousands of SNPs and a large population of families and offspring, enabled more reliable predictions of chromosomal rearrangements. The fact that our suggested rearrangements were validated by FISH analyses provides significant confidence for these predictions.

The Atlantic salmon genome seems to be remarkable in its tolerance to chromosomal rearrangements and plasticity. The ability of two individuals with different numbers of chromosomes to mate successfully may relate to the presence of pseudolinkage and residual tetrasomy in male salmonids [1,31,32] that may somehow help to prevent aneuploidy in the juveniles. Eggs and juveniles from the F1 and F2 generations of a cross made using one grandparent of each subspecies had high survival rates [33]. While our work describes the most common chromosome architecture of European and North American Atlantic salmon, the analysis utilized material collected from specific breeding programs and the results may not be universally representative of North American Atlantic salmon. Indeed, several other karyotypes have been reported for Atlantic salmon from Maine and Canada. For example, Roberts [34] reported a 2n of 56 or 57 in landlocked salmon from Maine, while Nygren [35] reported that salmon from Baie de Chaleur (Quebec) have a 2n of 58, similar to salmon from Sweden. Boothroyd [36] surveyed chromosomes from three river populations (Gaspe, Quebec; Miramichi, New Brunswick; and River Phillip, Nova Scotia) and reported a 2n of 56 for all. However it is not always clear whether these differences are true karyotypes or artifacts attributed to (1) the different tissues used for analysis [21,36,37], or (2) due to the fact that many sampled fish were hatchery culls already displaying deviant phenotypes [36]. Preparations of chromosomes for the FISH-analysis in this study, revealed heterozygosity of the fusions (ssa26/28 and/or ssa08/29) in different individuals, but all North American Atlantic salmon were homozygous for the ssa01p/23 translocation. These observations could explain the many differing reports on the North American Atlantic salmon karyotype. It would be interesting to conduct a survey of the karyotypes in different population of Atlantic salmon from North America, in particular documenting the distribution of the chromosomal rearrangements described in this report.

## Conclusions

Our comparison of the dense SNP linkage maps for Atlantic salmon derived from either side of the Atlantic Ocean identified major chromosomal differences between European and North American Atlantic salmon, which were confirmed by FISH analysis. Despite this, marker order is well conserved and a large proportion of the SNPs generated from European Atlantic salmon work are informative in the North American population. Thus, while gross structural differences are significant, the DNA sequence at the fine-resolution scale is remarkably conserved. This is a good indication that information from the International Cooperation to Sequence the Atlantic Salmon Genome [38], which is utilizing a European Atlantic salmon, can be transferred to salmon derived from the other side of the Atlantic Ocean.

## Methods

### Genetic linkage mapping

#### Mapping panel and family pedigree structures

The mapping panel consisted of two distinct sample groups. The first group comprised 160 parents and 1669 offspring from a Tasmanian aquaculture program, which was established from salmon originating from the River Philip, Nova Scotia in the 1960s. The second group comprised 38 parents and 107 offspring from an aquaculture program in Atlantic Canada, which were founded using salmon from the Saint John River, New Brunswick (Cooke Aquaculture Ltd). Within the 97 families from both groups, offspring represented a mixture of half-sibs and full-sibs. Where information from one parent was missing, the family was entered as half-sib family. For all samples, high molecular weight genomic DNA was isolated from fin-clips using commercially available kits. DNA was quantified, normalized and quality checked before genotyping.

#### Genotyping array

All 1974 samples were genotyped using a custom Atlantic salmon iSelect SNP-array manufactured by Illumina (San Diego, USA). This particular array is a newer version of the salmon array described by Bourett et al. [39], and was used for European Atlantic salmon linkage map construction [24]. The SNPs were sourced from public EST databases and resequencing of Norwegian aquaculture stocks. Of its 5,568 functional SNP assays 5,349 (96%) were present on the original array, making results comparable.

Because this is a non-standard array, a pre-existing cluster file was not available; therefore, automatic clustering was used to produce general SNP and sample statistics. However, the partially tetraploid nature of the Atlantic salmon genome means that some markers did not show standard diploid distribution and required manual cluster adjustment. For this purpose additional samples (480) from diverse geographic locations were included. This helped to ensure that all alleles were represented for each SNP and improved our confidence when re-clustering. Once the best possible clustering had been done, genotypes were exported to a database. Individuals and families were then examined for pedigree errors, and this resulted in a final set of 1,974 salmon being used for the linkage map construction.

#### Linkage map construction

Modified versions of the CRIMAP 2.4 software [40] were utilized for the linkage analysis following the procedure of Lien et al. [24]. The linkage analyses included all families from both Tasmanian and Saint John River aquaculture strains. Additionally, separate analyses were performed for markers located on linkage groups involving chromosomal differences between European and North American Atlantic salmon, but this did not reveal any significant differences in linkage concerning fusion points between the two populations. Graphical visualization of chromosome differences between Atlantic salmon from either side of the Atlantic Ocean was produced by a customized R-script.

### Fluorescence *in situ* hybridization (FISH)

#### Chromosome preparations

European Atlantic salmon (Mowi strain, from R. Devlin, Fisheries and Ocean Canada, West Vancouver Lab) weighing 2–5 kg were anaesthetized using MS222. The fish skin was cleaned with 70% EtOH and Kimwipes. Up to 2 mL of blood was aseptically drawn from the caudal vein of the fish using a sterile syringe inserted near the anal fin. The blood was collected into a Vacutainer tube containing heparin, gently mixed and transported at 4°C to the Davidson lab at Simon Fraser University.

A similar procedure was used to obtain blood from Atlantic salmon from the Aqua Bounty Canada transgenic broodstock in Prince Edward Island. The blood was collected early in the morning in Prince Edward Island and shipped at 4°C to the Davidson lab at Simon Fraser, arriving within 24 h.

The original transgenic Atlantic salmon was produced at Memorial University, St. John’s, Newfoundland [41], and the transgenic broodstock has been crossed with Saint John River, New Brunswick salmon for at least four generations (J. Buchanan personal communication to W.S. Davidson). The sub-species status of the original Newfoundland Atlantic salmon is unknown, but it has been shown that Newfoundland is a hybrid zone, containing Atlantic salmon with European as well as North American mtDNA haplotypes [42]. Although we originally suspected that the fish that were sampled correspond to a North American-European hybrid, backcrossed at least four times with North American Atlantic salmon, this does not appear to be the most parsimonious way to explain the observed karyotype; rather, it appears that these Atlantic salmon contain common polymorphisms for two chromosomal fusions.

The heparinized blood was thoroughly mixed with 5 mL of media L-15 (Gibco) in a 15 mL sterile plastic tube and placed on ice for 5 minutes. The diluted blood was then centrifuged at 1,200 rpm for 5 minutes at room temperature. After centrifugation, the buffy coat (containing lymphocytes) above the red blood cells was floated in plasma by a gentle stirring with a 1 mL pipette. The lymphocyte-enriched plasma was then collected in a new 15 mL sterile plastic tube. The plasma was centrifuged at 1,500 rpm for 5 minutes, and the resulting cell pellet was suspended in 5 mL of complete media L-15 containing 10% fetal bovine serum (FBS), 60 μg/mL of kanamycin sulfate, 1 x antibiotic-antimycotic solution (100 U/mL of penicillin, 100 μg/mL of streptomycin and 250 ng of amphotericin B), 25 μM of 2-mercaptoethanol and 18 μg/mL of phytohemagglutinin (PHA-W) and 100 μg/mL of lipopolysaccharide (LPS). The cells were cultured at 18°C in a culture tube slanting to an angle of about 30° with gentle daily mixing for 6 days. About 90 minutes before cell harvest, the lymphocyte culture was supplemented with 500 ng/mL colcemid. The cells were collected by centrifugation at 1,500 rpm for 5 minutes, and the supernatant was discarded. The cell pellet was suspended in 2 mL of 0.075 M KCl hypotonic solution for 20 minutes at 20°C. The hypotonic solution was slowly added to a volume of 2 mL. Then, 2 mL of fresh Carnoy’s fixative (3 methanol: 1 acetic acid) was added slowly. After centrifugation at 1,500 rpm for 5 minutes, the supernatant was discarded. The fixed cells were gently suspended in 3 mL of Carnoy’s fixative. The fixation step was repeated two more times, and then the cells were suspended 1–2 mL of Carnoy’s fixative. A microscope slide was exposed to hot water vapor at 73.5°C for 30 seconds.The cell suspension was immediately dropped on to the slide. After the slide surface became “grainy”, the slide was immediately exposed again to the hot water vapor at 73.5°C for 30 seconds. The slide was then quickly dried on a hot surface, which provided good chromosome spreading.

#### FISH analysis

FISH analyses were carried out using a modification of the procedure described in Li et al. [43]. BAC DNA, prepared using a large construct kit (Qiagen), was labeled with either SpectrumOrange (Vysis) or SpectrumGreen (Vysis) as recommended by manufacturers with minor modifications. Five hundred ng of extracted BAC DNA was mixed with 1.25 μL of SpectrumOrange or SpectrumGreen, 2.5 μL of 0.1 mM dTTP, 5 μL 0.1 mM dNTP mix, 2.5 μL 10 × nick translation buffer and 2.5 μL of nick translation enzyme in 25 μL. The reaction mixture was briefly mixed and centrifuged, and incubated in a PCR thermocycler at 15°C for 8 hours, followed by 70°C for 10 minutes, and then paused at 4°C. The probe size was determined by running a 1.3% agarose gel containing EtBr at 140 volts for 30 minutes together with a 100 bp ladder.

Hybridization of the fluorescent-labeled DNA probe with chromosome spreads was performed as suggested by the manufacture (Vysis) with minor modifications. Five μL of the nick translation reaction mixture of the BAC containing one microsatellite marker and 5 μL of the nick translation reaction mixture of the BAC containing another microsatellite marker, which were used to predict the chromosomal locations of the BACs [24] were mixed together with 2 μg of Atlantic salmon Cot-1 DNA and 2 μg of human placental DNA in a microcentrifuge tube. 0.1 volume of 3 M sodium acetate and 2.5 volumes of 100% EtOH were added to precipitate the DNA. The mixture was incubated at −80°C for 60 minutes and then centrifuged at 12,000 rpm for 30 minutes at 4°C to pellet the DNA. The supernatant was removed and the pellet was dried for 15 minutes at room temperature. The pellet was then suspended in 3 μL of dH2O and 7 μL of hybridization buffer by shaking at 250 rpm for 30 minutes at 37°C. The probe was denatured by heating at 80°C for 5 minutes and then chilled on ice for 1 minute. Then the probe was incubated at 37°C from 30 to 60 minutes prior to hybridization. The freshly made metaphase containing slides were treated with 2 × SSC for 30 minutes at 37°C, and then serial dehydrated in 70% EtOH, 85% EtOH and 100% EtOH, with each treatment 2 minutes. The hybridization area was marked using a tipped scribe. The slide was denatured in 70% formamide in 2 x SSC, pH 7.0-8.0, at 73°C for 3 minutes. Then the slide was serial dehydrated in −20°C 70% EtOH, 85% EtOH and 100% EtOH, with each treatment being 2 minutes, and then air-dried. Ten μL denatured probe was added to the slide, and a coverslip was immediately applied and sealed with rubber cement. The slide was put in a sealed humidified box at 37°C for 16 hours. Then the coverslip was removed together with the rubber cement seal, and the slide was immediately placed into a 73°C 0.4 × SSC/0.3% NP-40 wash solution in a hot water bath for 4 minutes, with several agitations per minute. The slide was then treated with the 2 × SSC/0.1% NP-40 wash solution at room temperature for 2 minutes. The slide was dried in the dark, and then 10 μL of DAPI antifade solution (Invitrogen) was applied to the slide. A multiphoton confocal microscope A1R MP (Nikon) was used to check the metaphase spreads. Laser 405 was used to detect the DAPI stain, laser 488 was used for the green dUTP labelling and laser 560 was used for the orange dUTP labelling.

## Competing interests

The authors declare that they have no competing interests.

## Authors’ contributions

SB: Participated in the linkage analysis, interpreted data and drafted the manuscript. JL: Carried out the FISH-analysis. MPK : Was responsible for genotyping, SNP filtering and assisted in finalizing the manuscript. EGB: Provided family material and assisted in finalizing the manuscript. SD: Provided family material and assisted in finalizing the manuscript. WSD: Was responsible for the FISH-analysis, helped draft and finalize the manuscript. SL: Conceived the study, provided financial support, was responsible for the linkage analysis and helped draft and finalize the manuscript. All authors read and approved the final manuscript.

## Animal ethics statement

Where possible and appropriate information regarding animals and research methods has been provided according to the ARRIVE guidelines.

## Funding

This work has received funding from the Norwegian Research Council (NFR) (grant number, 177036/S10), the CSIRO Food Futures Flagship Program, and the Natural Sciences and Engineering Research Council of Canada (NSERC) Strategic Grants Program.

## Supplementary Material

Additional file 1 ** Linkage map for North American Atlantic salmon.** The columns in the table are ID of the marker, North American Atlantic salmon chromosomes number, order of markers within each linkage group, female linkage map in cM, male linkage map in cM, number of meioses, dbSNP accession number and North American specific SNPs (marked with x). (TXT 203 kb) click here for file
